# Money talks: neural substrate of modulation of fairness by monetary incentives

**DOI:** 10.3389/fnbeh.2014.00150

**Published:** 2014-05-05

**Authors:** Yuan Zhou, Yun Wang, Li-Lin Rao, Liu-Qing Yang, Shu Li

**Affiliations:** ^1^Key Laboratory of Behavioral Science, Magnetic Resonance Imaging Research Center, Institute of Psychology, Chinese Academy of SciencesBeijing, China; ^2^Institute of Psychology, University of Chinese Academy of SciencesBeijing, China

**Keywords:** fairness, insula, lateral prefrontal cortex, normative decision, ultimatum game

## Abstract

A unique feature of the human species is compliance with social norms, e.g., fairness, even though this normative decision means curbing self-interest. However, sometimes people prefer to pursue wealth at the expense of moral goodness. Specifically, deviations from a fairness-related normative choice have been observed in the presence of a high monetary incentive. The neural mechanism underlying this deviation from the fairness-related normative choice has yet to be determined. In order to address this issue, using functional magnetic resonance imaging we employed an ultimatum game (UG) paradigm in which fairness and a proposed monetary amount were orthogonally varied. We found evidence for a significant modulation by the proposed amount on fairness in the right lateral prefrontal cortex (PFC) and the bilateral insular cortices. Additionally, the insular subregions showed dissociable modulation patterns. Inter-individual differences in the modulation effects in the left inferior frontal gyrus (IFG) accounted for inter-individual differences in the behavioral modulation effect as measured by the rejection rate, supporting the concept that the PFC plays a critical role in making fairness-related normative decisions in a social interaction condition. Our findings provide neural evidence for the modulation of fairness by monetary incentives as well as accounting for inter-individual differences.

“To attain to this envied situation, the candidates for fortune too frequently abandon the paths of virtue; for unhappily, the road which leads to the one, and that which leads to the other, lie sometimes in very opposite direction.”

—Adam Smith: The theory of moral sentiments

## Introduction

A unique feature of human beings is compliance with social norms even though this normative decision means curbing self-interest (Buckholtz and Marois, 2012), which is another characteristic of human beings (Hobbes, 1994; Miller, 1999). However, sometimes money talks. In other words, people sometimes prefer to pursue wealth at the cost of moral goodness. How human beings make a normative decision when facing a large monetary temptation within a social interaction context is an interesting question.

The ultimatum game (UG) nicely illustrates fairness, a salient, and elementary norm (Roth et al., 1991; Henrich, 2000), and provides a good paradigm for describing deviations from fairness-related normative choices. In this game, two players have to agree on how to split a sum of money (stake). Player A (the proposer) makes a proposal about how to divide the money. If player B (the responder) accepts this proposal, the suggested split is realized. If the responder rejects this proposal, neither of the two receives anything. In either case, the game is then over (Güth et al., 1982). If the responder acts in his or her own economic self-interest, s/he should accept the smallest amount of money offered. However, a key observation in this game has been that responders accept fair proposals that are close to an equal split and tend to punish proposers who offer unfair proposals (usually less than 20–30% of the total stake), even at a cost to themselves (Güth et al., 1982; Camerer, 2003). The unfair proposals are viewed as a violation of the fairness norm and the rejection of unfair proposals is, therefore, a normative decision (Buckholtz and Marois, 2012). During this decision process, the responders face two competing goals—a self-interest goal and a fairness goal. Implementation of this normative decision means that the responders succeed in curbing their self-interest goal, i.e., maximizing their economic gain by accepting the low offer, in order to pursue their fairness goal, i.e., punishing the proposer for the unfair offer by rejecting it.

Previous behavioral studies that used the UG showed deviations from the fairness-related normative decision as a result of high monetary incentives. At high stakes, responders tend to reduce the threshold below which they reject proposals (Straub and Murnighan, 1995; Hoffman et al., 1996; Slonim and Roth, 1998; Cameron, 1999; Munier and Zaharia, 2003; Bechler, 2013), although a few studies reported that stake size did not affect the rejection rate in the UG (Carpenter et al., 2005). This commonly-found deviation from the fairness-related normative choice at a high stake has been nicely accounted for by opportunity cost (Bechler, 2013), which indicates that, when a larger initial sum of money is used, the cost of rejecting an offer increases and the material utility of accepting an offer increases. However, the neural substrate underlying this deviation from the fairness-related normative choice when facing a high monetary temptation in the UG is still to be determined.

Previous neuroimaging studies, all of which used small stake sizes, have revealed a neural network involved with fairness-related normative choice. This network includes the dorsolateral prefrontal cortex (DLPFC), ventrolateral prefrontal cortex, insula, and other regions such as the medial prefrontal cortex, anterior cingulate cortex (ACC), and amygdala (Sanfey et al., 2003; Tabibnia et al., 2008; Guroglu et al., 2010, 2011; Baumgartner et al., 2011; Gospic et al., 2011). However, the neural substrate underlying deviations from the fairness-related normative choice in UG has rarely been investigated. Using brain stimulation techniques which temporally inhibit the function of the DLPFC, researchers identified a role for the right DLPFC in facilitating fairness-related normative decisions (van't Wout et al., 2005; Knoch et al., 2006a,b, 2008). By combining a brain stimulation technique with fMRI, a recent study verified that the right DLPFC and its functional communication with the posterior part of ventromedial prefrontal cortex play a causal role in facilitating a fairness-related normative decision in contexts in which fairness is in conflict with self-interest (Baumgartner et al., 2011).

The goal of the current study was to identify the neural substrates underlying deviations from the fairness-related normative choice caused by a monetary incentive. One Way to address this modulation of fairness by the monetary incentives is by within-experiment comparisons of brain activation under different monetary incentives, holding all else constant. We hypothesized that monetary incentives may modulate fairness and proposed several candidate regions for these psychological processes. Specifically, we proposed two candidate regions as the ones whose activations might be modulated by the monetary incentives on fairness. One is the lateral prefrontal cortex (PFC). A large stake size may amplify the self-interest goal, and thus more cognitive effort needs to be exerted to resolve the conflict between self-interest and fairness. A suggested role of the PFC, especially the DLPFC, in the UG is that its involvement reflects an integration-and-selection function, which includes the inhibition of a prepotent response and the selection of a specific response from all the possible response options by integrating information with context-specific rules about how to apply this information (Buckholtz and Marois, 2012). Therefore, we speculated that activation of the PFC would be modulated by the size of monetary incentives. The other region that might be modulated is the insula. We speculate that monetary incentives may influence fairness perception in the UG. The insula is important in encoding fairness, and distinct functional dissociations of insular subregions have been found in the UG. The anterior insular cortex is especially linked with a perception of “unfairness” because of its role in processing negative emotional reactions (Sanfey et al., 2003). The posterior insula is related to objective inequality, as indicated by showing stronger activation with more equal allocations (Wright et al., 2011). The mid-insula seems to play a role in integrating the context with inequality in the UG (Wright et al., 2011). Therefore, we speculated that activation of the insula would also be modulated by monetary incentives and also expected to find dissociable modulation patterns in insular subregions. By investigating the modulation of fairness by the size of a monetary incentive, we expect to better understand the functioning of the normal normative decision.

## Materials and methods

### Participants

A total of 28 participants (15 females and 13 males, mean age = 25.07 ± 3.35 years, mean years of education = 17.32 ± 1.72) were recruited through advertisement. All participants were in good health with no previous history of psychiatric or neurological disease and were screened for standard magnetic resonance safety criteria. All participants gave written informed consent. The study was approved by the Institutional Review Board of the Institute of Psychology, the Chinese Academy of Sciences.

### Experimental design and task

#### General procedure

Prior to the scanning session, the participants received instructions explaining the rules of the UG that they would be performing inside the scanner, and each participant was required to complete a series of test questions after reading the instructions to verify their comprehension. During the scanning, the participants acted as responders in a series of rounds of the UG, during which they might play with a computer or with a person. After scanning, the participants rated the fairness of all the proposals presented in the UG task on a Likert scale of 1 (very unfair) to 7 (very fair). The goal of this questionnaire was to evaluate the participants' subjective fairness judgment. To increase the degree of their involvement in this experimental situation, they also made proposals with different stake sizes as proposers and were told that their proposals would be used in a subsequent study.

#### UG task description

**Figure 1:**
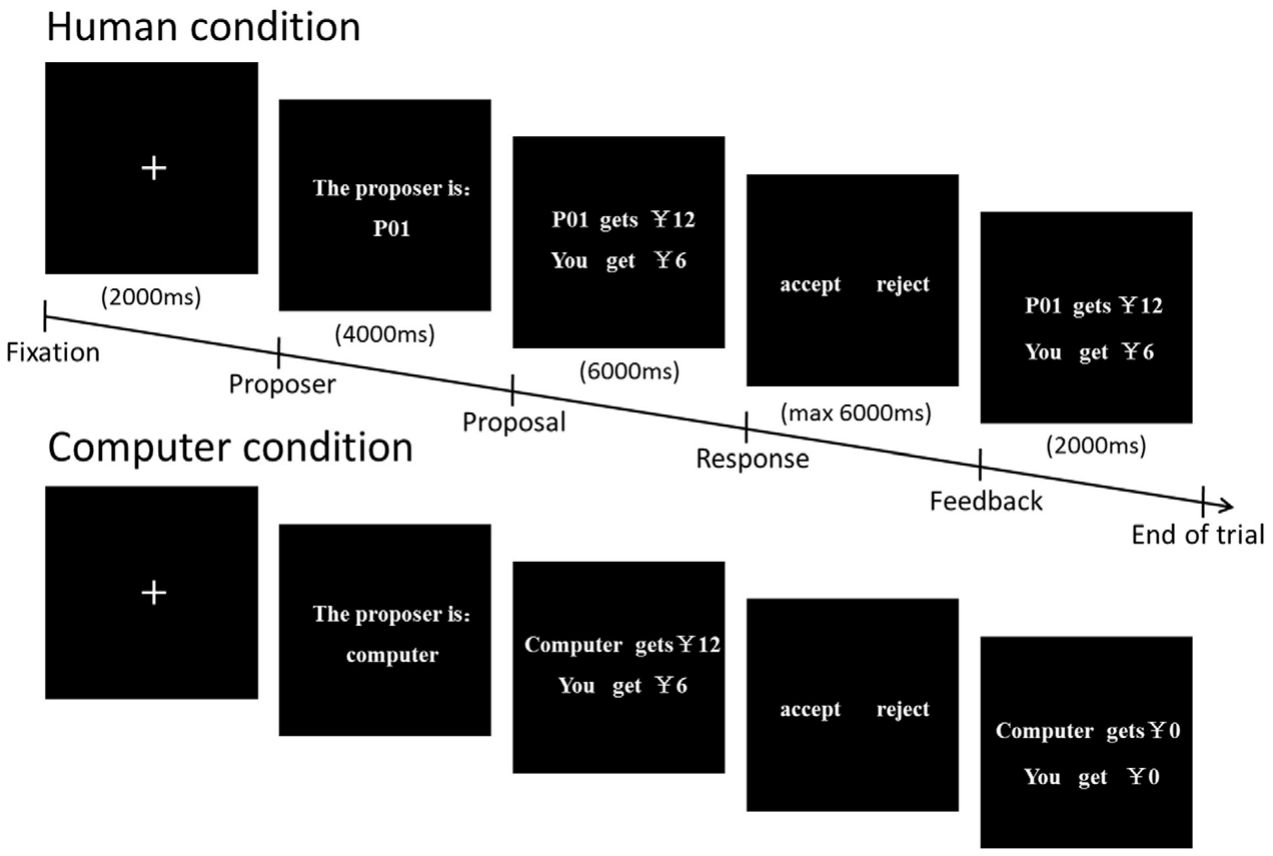
**Diagram illustrating the structure of a single round of the ultimatum game**.

While they were in the scanner, each participant played the role of a responder, receiving 24 rounds of monetary proposals. Each round had five phases (Figure 1). Each round began with a 2 s preparation interval. The participant then saw the number of the proposer (e.g., P01) or computer for 4 s. Next, the offer proposed by the partner was displayed for a further 6 s. Afterwards, the participant was asked to respond by pressing one button to accept and another to reject the offer with a maximum of 6 s for this process. Then the result of the choice was showed for 2 s. Half of the proposals were from the human partners and the other half were from the computer partners. In the human condition, the participants were informed that proposals from real persons had been submitted by previous participants and that in each round their partners would be different (a single-shot game). To avoid uncontrolled associations, the human proposers were represented by alphanumerical codes, not by their photos or by their real names in accordance with a previous study (Halko et al., 2009). In the computer condition, the participants were told that the proposals from the computer were randomly generated by a computer program. In reality, all the proposals were pre-set by the experimenter. Therefore, the computer condition was similar to the human condition in terms of fairness and self-interest, except for the fact that there was no potential for social interaction in the computer condition. Offering the computer condition provided us with an opportunity to investigate whether people modulate their response to fairness by the size of a monetary incentive only in a social interaction context.

We orthogonally manipulated fairness and monetary incentives by varying both the proposal amount and the stake size across the rounds (Table 1). Two kinds of proposals were provided, and each one fell into one of the two “fairness” categories: 50% of the stake (fair) or 20% of the stake (unfair). We used only one unfair level, i.e., proposals of 20% of the stake, referring to previous studies (Falk et al., 2003; Radke et al., 2012). The high monetary incentives ranged from ¥400(≈$64) to ¥600(≈$96) and the low ones from ¥4(≈$0.64) to ¥6(≈$0.96). In different rounds, the same proposal amount (e.g., ¥400) could represent a larger percentage of the total stake (50%) and could, therefore, be fair, or a smaller percentage of the total stake (20%) and could, therefore, be unfair. This design controlled for potential confounding effects of the proposal magnitude. Therefore this study was a 2 × 2 × 2 design, with proposer type (human, computer), stake size (high, low), and fairness (50%, 20%) as within-subjects factors (Table 1). There were a total of 24 trials in our experiment design, so there were three trials in each condition. To encourage participants to make real decisions, it was emphasized that in addition to a fixed amount for participation they would be paid according to their choices in the game.

**Table 1:** **Types of offers**.

	**Fair (50%) Proposer–Responder**	**Unfair (20%) Proposer–Responder**
High	¥400–¥400	¥1600–¥400
	¥500–¥500	¥2000–¥500
	¥600–¥600	¥2400–¥600
Low	¥4–¥4	¥16–¥4
	¥5–¥5	¥20–¥5
	¥6–¥6	¥24–¥6

### fMRI data acquisition

The stimuli were presented with E-prime software (Psychology Software Tools, Pittsburgh, PA) on a personal computer, back-projected onto a screen using a liquid crystal display projector, and viewed by participants through a mirror mounted on the MRI head coil. The MRI images were acquired with a 3.0 Tesla Siemens MRI scanner. Whole-brain functional scans were collected in 32 axial slices using an echo-planar imaging (EPI) sequence (repetition time = 2000 ms, echo time = 30 ms; flip angle = 90°, matrix = 64 × 64; field of view = 220 × 220 mm^2^; slice thickness = 3 mm; slice gap = 1 mm). Before the task fMRI, we also acquired a 6-min resting-state scan, which was composed of 180 volumes. These data were not used in this study.

### fMRI preprocessing and analyses

#### Image preprocessing

Image preprocessing was performed using statistical parametric mapping (SPM5, Wellcome Department, London, UK) running on a Matlab 7 platform (MathWorks, Natick, MA). The preprocessing included slice time correction, realignment, normalization, and resampling to 3 × 3 × 3 mm^3^, and smoothing using an 8-mm full-width-at-half-maximum Gaussian kernel.

#### Category analyses

A general linear model (GLM) with a 2 (fairness) × 2 (proposer type) × 2 (stake size) factorial design matrix was constructed to detect the brain activation of each participant during the proposal epochs. Specifically, a GLM was defined for each participant. These models included eight regressors which modeled the BOLD response to the 6 s proposal epoch: fair proposal from a human partner, unfair proposal from a human partner, fair proposal from a computer partner and unfair proposal from a computer partner for each of the high and low stake sizes. Additionally, six motion parameters obtained by realignment were used as nuisance variables. Each regressor was convolved with a canonical hemodynamic response function. High-pass filtering (cutoff frequency = 128 s) was used to remove low-frequency noise. The resulting GLM was corrected for temporal autocorrelations using a first-order autoregressive model. First-level contrasts were performed for each experimental condition of the factorial design described above. A second-level random effect analysis was completed by performing an ANOVA on these contrasts. In the second-level analysis, we used a mask that was created with the wfu_pickatlas tool (Tzourio-Mazoyer et al., 2002) in SPM5. The mask was the composite of the following 16 bilateral regions of interest (ROI): superior frontal cortex, middle frontal cortex, inferior frontal operculum, inferior frontal triangularis, superior medial frontal cortex, superior orbitofrontal cortex, medial frontal cortex, medial orbitofrontal cortex, inferior orbitofrontal cortex, rectus, ACC, insula, caudate, thalamus, putamen, and amygdale. These ROI were selected based on the previous literature (Sanfey et al., 2003; Tabibnia et al., 2008; Guroglu et al., 2010, 2011; Baumgartner et al., 2011; Gospic et al., 2011). Significant activations of interest were identified with voxelwise *p* < 0.005 in conjunction with clusterwise *p* < 0.05 to perform a multiple comparisons correction. If an interaction effect was statistically significant, simple effect analyses were performed using the averaged effect size extracted from the clusters with significant interaction effects using SPSS v17.0.

The previous literature has suggested different roles for different insular subregions in fairness-related decisions (Sanfey et al., 2003; Wright et al., 2011). Thus, it is possible that a dissociable modulation effect of fairness by monetary incentives may be observed in insular subregions. To test this hypothesis, we parceled the insular clusters into the ventral anterior, dorsal anterior and mid-posterior clusters by intersecting the insular subregions template (*k* = 3 solutions) (Kelly et al., 2012) with those of our insular clusters which showed significant interaction effects. Then, we performed simple effect analyses on each of the resulting insular subregions separately.

#### Multiple regression analysis

In order to investigate the neural correlates of inter-individual differences in the behavioral modulation effect, we defined an index for the behavioral modulation effect. This index was the interaction between fairness and stake size in rejection rate, i.e., rejection rate [high_(unfair−fair)_ − low_(unfair−fair)_]. Because the rejection rate for fair proposals with high stake sizes was similar to that for fair proposals with low stake sizes, the index reflected the extent of the difference between the rejection rate for unfair proposals with high stake sizes and that for unfair proposals with low stake sizes. According to previous studies in which the rejection rate for unfair proposals decreased with increased stake sizes (Straub and Murnighan, 1995; Hoffman et al., 1996; Slonim and Roth, 1998; Cameron, 1999; Munier and Zaharia, 2003; Bechler, 2013), a negative value of this index indicates a deviation from the normative decision, a positive value reflects a normative decision, and zero reflects indifference to the changes of stake size.

Multiple regression analysis was performed with the behavioral modulation index as the covariate variable and the neuroimaging contrasts from the modulation effect [high_(unfair−fair)_ − low_(unfair−fair)_] as the dependent variable. The mask that was used in the category analysis was also used for the multiple comparison correction. Significant effects were identified with voxelwise *p* < 0.005 in conjunction with a clusterwise *p* < 0.05.

## Results

### Behavioral results

**Figure 2:**
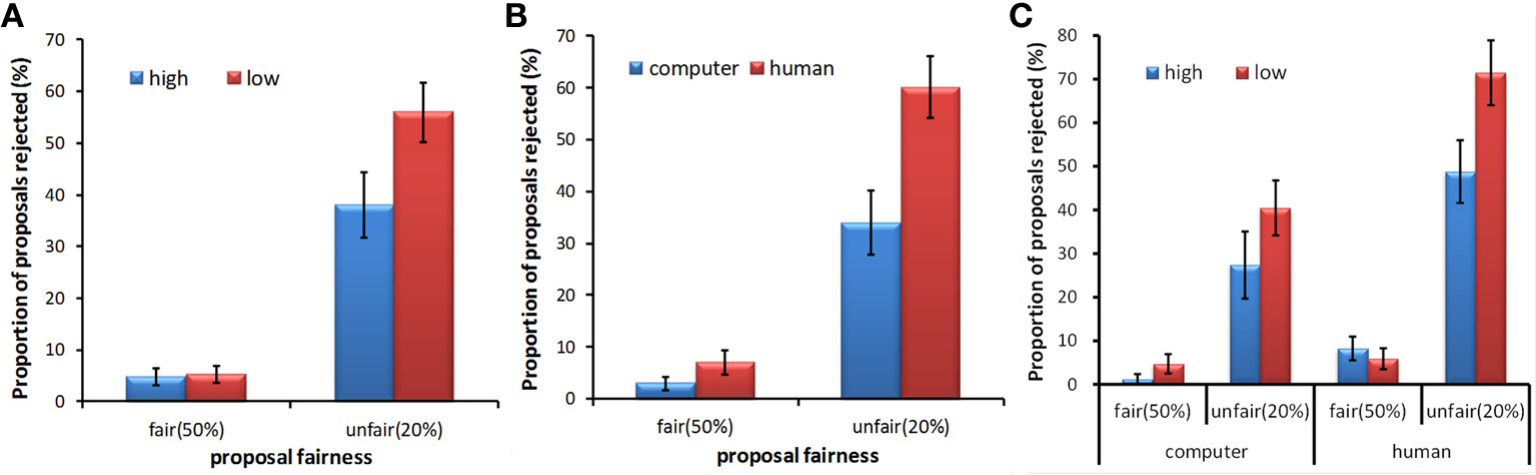
**Mean rejection rates as a function of proposal fairness for different stake sizes (A), proposer types (B), and the interaction among these three factors (C)**. Error bars indicate standard errors of the mean.

The rejection rate was investigated by a repeated measures ANOVA, exploring the main effects of fairness (50%, 20%), proposer type (human, computer), stake size (high, low), and the interaction between these factors. Mauchly's test of sphericity showed that the sphericity assumption was met (all *p*s > 0.05). Significant main effects of fairness [*F*_(1, 27)_ = 65.34, *p* < 0.001, partial η^2^ = 0.708], stake size [*F*_(1, 27)_ = 7.43, *p* = 0.011, partial η^2^ = 0.216] and proposer type [*F*_(1, 27)_ = 18.22, *p* < 0.001, partial η^2^ = 0.403] were found. These main effects separately indicated that unfair proposals (*M* = 0.47, *SD* = 0.28) were more often rejected than fair ones (*M* = 0.05, *SD* = 0.07), proposals from humans (*M* = 0.34, *SD* = 0.18) were more often rejected than those from computers (*M* = 0.18, *SD* = 0.17), and proposals with a low stake size (*M* = 0.31, *SD* = 0.17) were more often rejected than those with a high stake size (*M* = 0.21, *SD* = 0.17). The interaction between fairness and stake size was significant [*F*_(1, 27)_ = 8.68, *p* = 0.007, partial η^2^ = 0.243]. A *post-hoc* pairwise least significant difference (LSD) test indicated that when the proposals were unfair, the rejection rates for proposals with a low stake size (*M* = 0.56, *SD* = 0.31) were significantly higher than those for proposals with a high stake size (*M* = 0.38, *SD* = 0.33) (Figure 2A). The interaction between fairness and proposer type was also significant [*F*_(1, 27)_ = 13.52, *p* = 0.001, partial η^2^ = 0.334]. A *post-hoc* pairwise LSD test indicated that the rejection rates for proposals from human partners (*M* = 0.60, *SD* = 0.32) were significantly higher than those for proposals from computer partners (*M* = 0.34, *SD* = 0.33) when the proposals were unfair (Figure 2B). There were no significant differences in rejection rates when the proposals were fair for these interactions. No significant interaction between stake size and proposal type was found. There was a trend toward significance in interaction among the three factors [*F*_(1, 27)_ = 3.09, *p* = 0.09, partial η^2^ = 0.103]. A *post-hoc* analysis revealed that the rejection rate for the unfair proposals with a high stake size was lower than that for the unfair proposals with a low stake size in the human condition (*p* = 0.012), whereas this difference only showed a trend toward significance in the computer condition (*p* = 0.062). In addition, we found no differences between the rejection rate for the fair proposal with a high stake size and that for the fair proposal with a low stake size in either the human condition or the computer condition (*p*s > 0.05) (Figure 2C).

A repeated measures ANOVA that considered the stake size and fairness categories, was conducted to examine the subjects' fairness judgments of different proposals. Mauchly's test of sphericity showed the sphericity assumption was met (*p* > 0.05). The results showed a significant main effect of the fairness category on the participants' ratings of the fairness of the proposals [*F*_(1, 27)_ = 369.59, *p* < 0.001, partial η^2^ = 0.932], but we did not find a significant main effect of stake size. The interaction effect of the fairness category and stake size was marginally significant [*F*_(1, 27)_ = 3.61, *p* = 0.068, partial η^2^ = 0.118]. A *post-hoc* pairwise LSD test indicated that fair proposals with a low stake size (*M* = 6.45, *SD* = 0.79) were rated as marginally significantly (*p* = 0.067) less fair than fair proposals with a high stake size (*M* = 6.57, *SD* = 0.60). When the proposals were unfair, we found no significant differences in fairness ratings between proposals with a high stake size and proposals with a low stake size.

### Brain activations influenced by fairness, stake size, and proposer type

**Figure 3:**
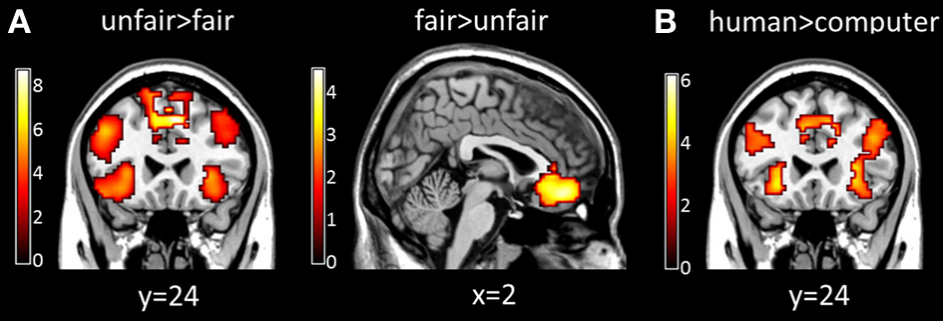
**Brain activations influenced by fairness and proposer type at proposal presentation. (A)** Maps of the *t* statistics for the contrast [unfair > fair] showing activation of the bilateral insula, ACC, and lateral PFC, and for the contrast [fair > unfair] showing activation of the medial prefrontal cortex. **(B)** Map of the *t* statistic for the contrast [human > computer] showing activation of the bilateral insula, ACC, medial prefrontal cortex and lateral PFC. Abbreviations: ACC, anterior cingulate cortex; PFC, prefrontal cortex.

A significant main effect of fairness was found in several brain regions. Specifically, the bilateral insula, ACC, lateral PFC showed greater activation for unfair proposals than for fair proposals, whereas the medial prefrontal cortex showed greater activation for fair proposals than for unfair proposals (*p* < 0.05, cluster-level correction) (Table 2 and Figure 3A). A significant main effect of proposer type was also found, in which the bilateral insula, ACC, medial prefrontal cortex, lateral PFC were more activated in the human proposer condition than in the computer proposer condition (*p* < 0.05, cluster-level correction) (Table 2 and Figure 3B). No significant main effect of stake size was found (*p* < 0.05, cluster-level correction).

**Table 2:** **Results using a factorial model for analysis of the fMRI data**.

**Cluster size**	**Hemisphere**	**Brain region**	**BA**	**MNI coordinates**	**Peak *T*-value**
**FAIR > UNFAIR**					
369	Bilateral	Medial frontal gyrus/anterior cingulate	11/32/10	6, 45, −12	4.64
**FAIR < UNFAIR**					
2705	Bilateral	Middle frontal gyrus/inferior frontal gyrus/superior frontal gyrus/medial frontal gyrus/insula	6/10/9/8	9, 24, 42	8.66
744	Right	Middle frontal gyrus/inferior frontal gyrus	9/8/6/46	42, 33, 27	5.43
377	Right	Middle frontal gyrus	10	42, 51, −12	4.93
209	Right	Inferior frontal gyrus/insula	47/13	36, 21, −3	5.41
**HUMAN > COMPUTER**					
634	Right	Middle frontal gyrus/inferior frontal gyrus/insula	9/46/47	45, 9, 30	4.76
252	Left	Inferior frontal gyrus/insula	47/10	−27, 24, −6	4.65
619	Left	Middle frontal gyrus/inferior frontal gyrus	6/9	−45, 9, 24	6.08
392	Left	Medial frontal gyrus/anterior cingulate	32/9	−9, 27, 33	4.35
**HIGH_(UNFAIR–FAIR)_ > LOW_(UNFAIR–FAIR)_**					
247	Left	Insula/putamen	13	−33, −6, 12	4.28
237	Right	Insula/putamen	13	36, −9, 9	4.70
268	Right	Middle frontal gyrus/inferior frontal gyrus	8/9/46	48, 9, 18	3.88
**HIGH_(UNFAIR–FAIR)_ > LOW_(UNFAIR–FAIR)_ WITHIN HUMAN**					
135	Left	Insula	13	−36, −18, 15	4.01
186	Right	Insula	13	36, −6, 3	4.35
56	Left	Putamen	–	−15, 6, −6	3.81
21	Right	Inferior frontal gyrus	46	54, 39, 9	2.96
20	Right	Superior frontal gyrus	8	15, 39, 48	3.12

### Brain activations influenced by the interaction of fairness and stake size

**Figure 4:**
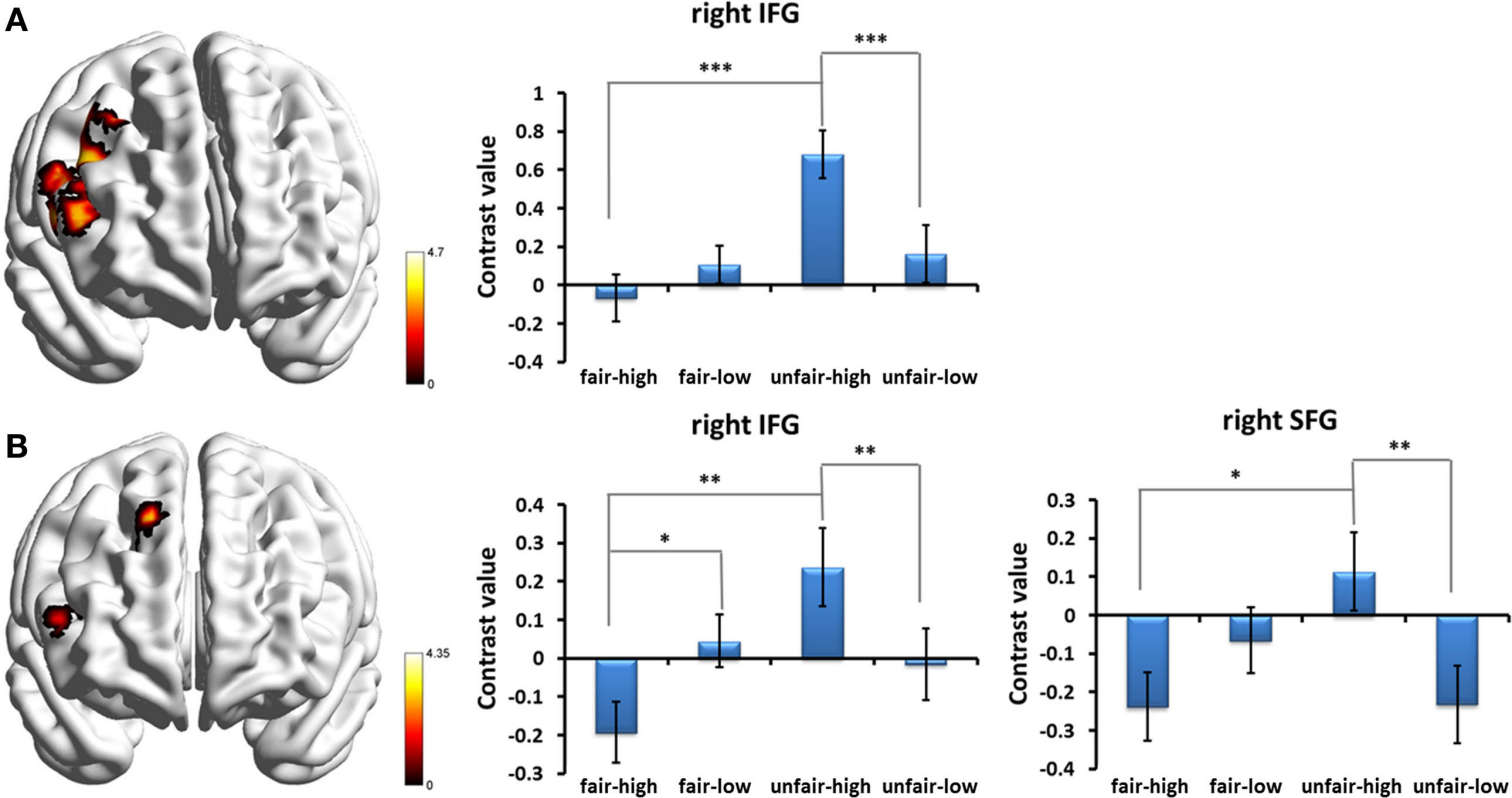
**Prefrontal cortices influenced by the interaction between fairness and stake size and simple effects of the interaction effects in (A) all the proposal conditions and (B) the human proposal condition**. Abbreviations: IFG, inferior frontal gyrus; SFG, superior frontal gyrus. ^*^p < 0.05, ^**^p < 0.01, ^***^p < 0.001. Error bars indicate standard errors of the mean.

Given that our primary goal was to test the influence of stake size on fairness, an interesting contrast was computed by the [high_(unfair−fair)_ − low_(unfair−fair)_] contrast. The bilateral insula extending to the adjacent striatum and the right middle/inferior frontal gyrus (IFG) were found to show significant contrast (*p* < 0.05, cluster-level correction) (Table 2). No regions survived the reverse contrast. An examination of the simple effects showed that the activation in the frontal cluster during unfair proposals was significantly stronger than during fair proposals when the stake size was high [*post-hoc* pairwise comparison, *F*_(1, 27)_ = 24.82, *p* < 0.001, partial η^2^ = 0.479], and a similar trend was found while the stake size was low but did not meet statistical significance. In addition, the activation in the frontal cluster for high monetary proposals was significantly stronger than for low monetary proposals during the unfair proposal condition [*F*_(1, 27)_ = 19.33, *p* < 0.001, partial η^2^ = 0.417] (Figure 4A). When we separated the human from the computer condition to examine the interaction of stake size and fairness, a similar pattern of interaction but with smaller clusters within those regions was found in the human condition (Table 2 and Figure 4B). No regions were influenced by the interaction between stake size and fairness in the computer condition (*p* < 0.05, cluster-level correction).

**Figure 5:**
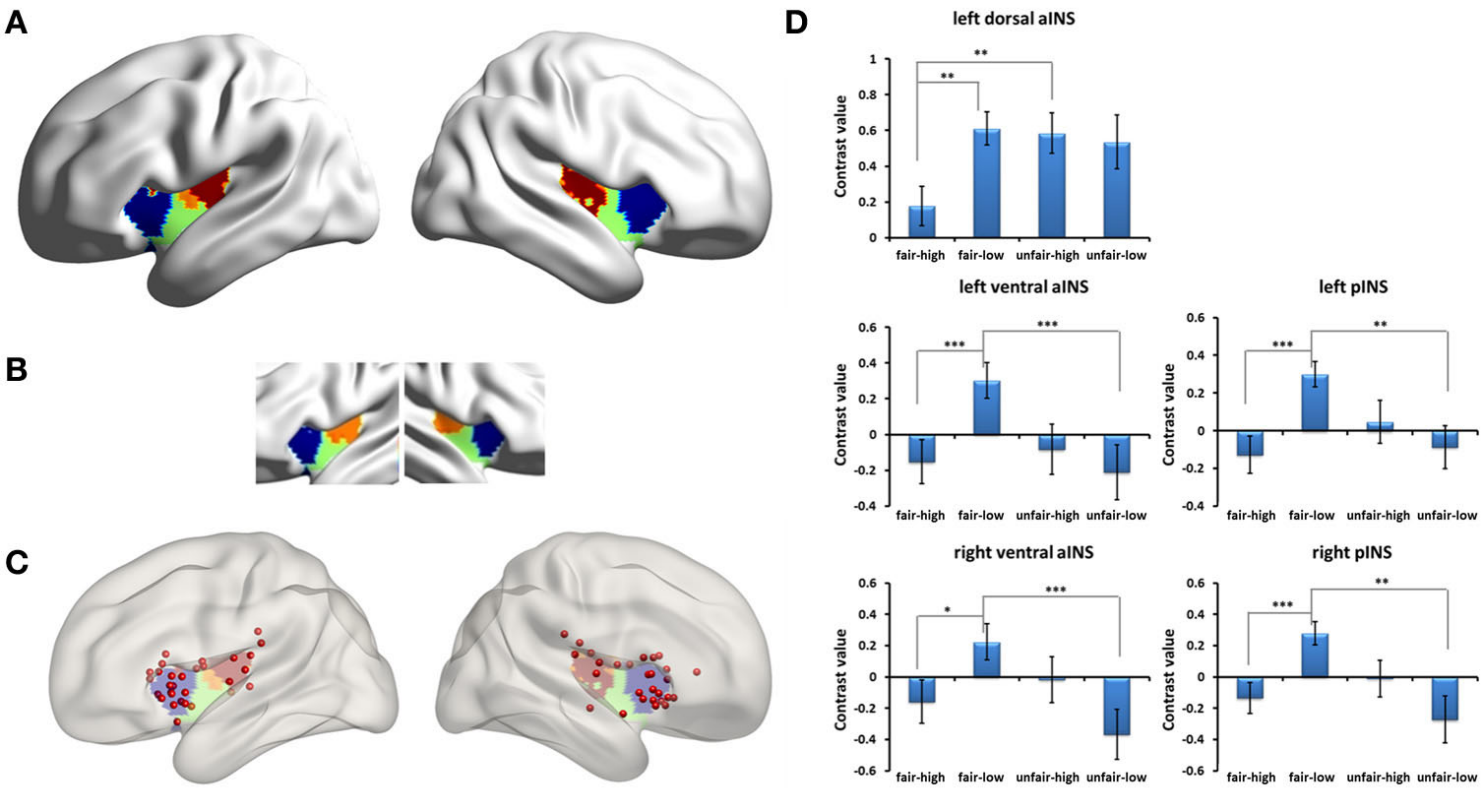
**Insular subregions influenced by the interaction of fairness and stake size for all the proposals. (A)** Insular subregions intersected by Kelly's template. **(B)** Kelly's insular subregions template (*k* = 3 solutions). **(C)** Locations of the reported insular clusters on Kelly's template. For each location, please see Table S3 in the Supplementary Materials. **(D)** Simple effects of the modulation of fairness by stake size in insular subregions. Abbreviations: aINS, anterior insula; pINS, posterior insula. ^*^p < 0.05, ^**^p < 0.01, ^***^p < 0.001. Error bars indicate standard errors of the mean.

Due to previous studies that indicated that the insular subregions functioned differently when responders faced fair and unfair proposals (Sanfey et al., 2003; Wright et al., 2011), we divided the clusters that included the bilateral insular cortices into subregions by intersecting them with an insular subregions template (*k* = 3 solutions) (Kelly et al., 2012). When we did not separate the human condition from the computer condition, we found significant activation in the left dorsal-anterior insula, the bilateral ventral-anterior insula, and the bilateral mid-posterior insula and examined the simple effects of the modulation of fairness by stake size in these insular subregions. The activation in the left dorsal-anterior insula cortex was significantly stronger for unfair proposals than for fair proposals when the stake size was high [*F*_(1, 27)_ = 12.04, *p* = 0.002, partial η^2^ = 0.308], but there were no differences when the stake size was low. In addition, the activation in the left dorsal-anterior insula cortex for low monetary proposals was significantly stronger than for high monetary proposals during the fair proposal condition [*F*_(1, 27)_ = 15.53, *p* = 0.001, partial η^2^ = 0.365]. The activations in the bilateral mid-posterior insula cortices were significantly stronger for fair proposals than for unfair proposals when the stake size was low (*post-hoc* pairwise comparison, *Fs* > 14, *ps* = 0.001), but there were no differences when the stake size was high. In addition, the activations in the bilateral mid-posterior insula cortices for low monetary proposals were significantly stronger than for high monetary proposals during the fair proposal condition (*Fs* > 17, *ps* < 0.001). The activations of the bilateral ventral-anterior insula cortices were similar to those of the mid-posterior insular cortices (Figure 5). When we separated the human from the computer condition, a similar pattern of interaction in the insular subregions was found in the human condition (Figure S1). Because the clusters also included the striatum, we divided the clusters into subregions by intersecting them with the striatum three subregions template (http://fsl.fmrib.ox.ac.uk/fsl/fslwiki/Atlases/striatumconn) in order to examine the simple effect of each striatal subregion (for details, please see Figure S2 and other Supplementary Materials).

### Neural correlates of inter-individual differences in the behavioral modulation effect

**Figure 6:**
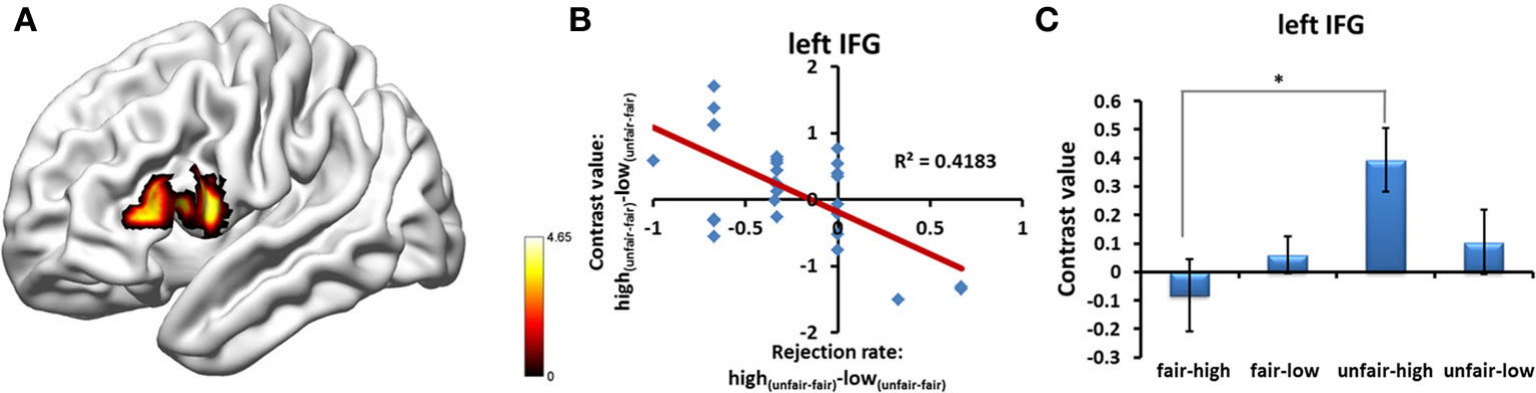
**(A)** Regions showing the neural correlates of inter-individual differences in the behavior modulation effect of the rejection rate in the human proposal condition. **(B)** Significant negative correlation was found in the left IFG between the bold signal of the [high_(unfair-fair)_ − low_(unfair-fair)_] contrast and the rejection rate of the [high_(unfair-fair)_ − low_(unfair-fair)_] proposals. **(C)** Significant interaction effect of fairness^*^stake size was found in the left IFG for participants who deviated from the fairness norm. Abbreviations: IFG, inferior frontal gyrus. ^*^p < 0.05. Error bars indicate standard errors of the mean.

Multiple regression analysis was performed to determine the regions whose BOLD signal change, which was detected using the [high_(unfair−fair)_ − low_(unfair−fair)_] contrast, varied with the modulation effect on the rejection rate. Because the pattern of the interaction between stake size and fairness in the human condition was different from that in the computer condition, as shown by the abovementioned analyses, in the main text we only showed the results obtained by separating the human proposals from the computer proposals when computing inter-individual indices (for the results obtained by combining the human proposals with those of the computer proposals, please see supplementary text and Figure S3). Significantly negative correlations were found in the left IFG (MNI coordinate = [−51, 15, 15]; *p* < 0.05, cluster-level correction) in the human condition (Figure 6 and Table S1), which suggests that a greater IFG signal for high_(unfair–fair)_ vs. low_(unfair–fair)_ stakes predicts a significant deviation from the fairness-related normative decision (i.e., a reduced behavioral rejection rate for an unfair proposal with a high stake size). No regions were found in the computer condition.

In order to take into consideration inter-individual differences, we analyzed those participants who showed a deviation from the fairness-related normative decision in the human condition (*n* = 17). By extracting the value of the mean of the interaction effect, the cluster in the left IFG showed a significant interaction effect (left IFG: *t* = 2.79, *p* = 0.013). We also conducted a one-sample t-test to examine the voxel-wise interaction effect contrast in these participants. The bilateral insula and IFG showed significant interaction effects (Table S2).

## Discussion

The goal of this study was to explore the neural substrates of the influence of monetary incentives on fairness. One important characteristic of our study was that we designed a computer condition, which shared many features with the human condition (e.g., fair perception, self-interest), but in which there were no social interactions. The computer condition provided us with an opportunity to investigate whether modulating the monetary incentive influences the perception of fairness only in a social interaction context. By distinguishing fairness level from offer amount, at the behavioral level we found that the magnitude of the stake size significantly modulated the rejection rates. Specifically, the rejection rates for unfair proposals with a high stake size were significantly lower than those for unfair proposals with a low stake size, a finding that was consistent with previous reports (Straub and Murnighan, 1995; Hoffman et al., 1996; Slonim and Roth, 1998; Cameron, 1999; Munier and Zaharia, 2003; Bechler, 2013). This behavioral deviation from the normative decision was reflected in their brain activation. As hypothesized, the fairness-related activations of the bilateral insular cortices and the right lateral PFC were modulated by the monetary incentives although different simple effects were identified. These modulation effects were only observed in the human condition, not in the computer condition, indicating that the modulation effect of fairness by monetary incentives only exists in social interaction situations. When we linked inter-individual differences in this behavioral modulation effect with brain activation, the left IFG was found to play a critical role in making the fairness-related normative decision in the social interaction condition.

### Modulation effects in the frontal cortices

The activation in the right DLPFC during the UG is often considered to reflect the involvement of cognitive control (Sanfey et al., 2003; Knoch et al., 2006a,b, 2008; Knoch and Fehr, 2007; Brune et al., 2012). However, there are different hypotheses about the psychological processes behind the activation of the right DLPFC during the UG. Some of them are partially divergent. For example, one view is that, in order to implement culturally acquired fairness norms, the right DLPFC is necessary to control the selfish motives of resource maximization (Knoch et al., 2006a,b, 2008; Knoch and Fehr, 2007). Another view is that the right DLPFC is involved in suppressing emotion-driven prepotent responses to perceived unfairness (Brune et al., 2012). An integration-and-selection hypothesis of the right DLPFC can be used to reconcile these divergent perspectives (Buckholtz and Marois, 2012). Specifically, the right DLPFC's involvement in the UG reflects an integration-and-selection function, which includes the inhibition of a prepotent response and the selection of a specific response from all the possible response options by integrating the current information with context-specific rules about how to apply this information (Buckholtz and Marois, 2012). According to this hypothesis, we speculated that in the context of an unfair proposal with a high stake size, such as that in the current study, if the self-interest impulse was the prepotent response, then the right DLPFC could be expected to be activated to inhibit this self-interest impulse, which led to an increased rejection rate. If the emotional (rejection) impulse was the prepotent response, in order to control the emotional impulse induced by unfair proposals, the right DLPFC could be expected to be activated, which would then lead to a reduced rejection rate. We found that the right DLPFC showed the modulation effect, as evidenced by an increased activation when facing an unfair proposal with a high stake size, in which lower rejection rate was observed. This result is consistent with the latter speculation.

In addition, we noted that the interaction effect in the lateral PFC was only seen when the proposals were from a human rather than a computer, which suggests a particular role of this region in a social interaction context. When a human made the proposal, the activations of both the right IFG and the right SFG were modulated by monetary incentives on fairness. However, there may be different explanations for the results from these two regions. The modulation effect in the right IFG may be accounted for by cognitive control for emotion-driven prepotent responses to perceived unfairness, based on previous evidence from neurophysiological, lesion, and task-based functional neuroimaging studies which suggest that cognitive control is associated with the right IFG (for a review, see Aron et al., 2004). The modulation effect in the right SFG may be attributed to emotional reappraisal. A recent UG study found that down-regulation strategies activated the right SFG (Grecucci et al., 2013). Monetary incentives have been found to decrease negative emotions (Zhou et al., 2009). Therefore, it is possible that large monetary incentives in the UG task may have played a role similar to that of an emotional down reappraisal by down-regulating the negative emotion, inducing strong activation in the right SFG, and thus making the participant more likely to accept an unfair offer with a high stake size.

Although previous studies have implicated the frontal cortices in UG behavior (such as Sanfey et al., 2003), the high stake size in the present study may have amplified the process of evaluation when the participants were making their decisions. The fact that the activation of the lateral PFC increased when facing an unfair proposal with a high stake size further clarifies the role of the frontal cortex in normative decision making. This role may well be to process the multiple motives and integrate them with emotional reactions to produce a context-appropriate decision (Buckholtz and Marois, 2012).

### Dissociable modulation effects in the insular subregions

In the present study, we found a dissociable pattern of the magnitude effect in the insular subregions. The insula is a cytoarchitectonically diverse cortical region and has been proposed as a nexus of sensory, somatic, interoceptive, cognitive, and emotional processing (Craig, 2009, 2011). These functions have been mapped into different subregions of the insula (Kurth et al., 2010; Kelly et al., 2012). In the context of the UG task, distinct fairness-related processes are expressed in segregated regions of the insula (Wright et al., 2011). Our findings also indicate that the distinct fairness-related processes expressed in the insula are modulated by monetary incentives.

The activation of anterior insula in the UG has often been suggested as resulting from the negative emotion triggered by unfairness (such as Sanfey et al., 2003). However, based on findings of functional differentiation in the insula (Kurth et al., 2010; Kelly et al., 2012), a recent study proposed a more attractive opinion that the anterior insula integrates information about modality-specific feelings with cognitive processes, individual preferences, and contextual information in order to promote fairness-related behavior (Corradi-Dell'Acqua et al., 2013). Our findings support this idea. By closely examining the locations of these insular clusters in various sources (Sanfey et al., 2003; Tabibnia et al., 2008; Chang and Sanfey, 2009; Halko et al., 2009; Guroglu et al., 2010, 2011; Gospic et al., 2011; Kirk et al., 2011; Wright et al., 2011; Harle and Sanfey, 2012; Harle et al., 2012; Kim et al., 2012; Corradi-Dell'Acqua et al., 2013; Grecucci et al., 2013; Guo et al., 2013), we found that the reported peak coordinates of the anterior insular clusters located in the dorsal-anterior part of the insula according to Kelly's template are most associated with cognition, unlike those of the ventral-anterior insula which are most associated with emotion (Table S1 and Figure 5C). Similarly, we found a significant modulation effect in this dorsal-anterior portion of the insula, not in the typical ventral-anterior insula. A further analysis showed that the significant modulation effect in the dorsal-anterior insula seems to have resulted from increased activity in connection with the fair proposal in the low stake size. Combining these neuroimaging findings with our behavioral finding from the fairness judgment scores that indicated that the fair proposal with a low stake size seemed less fair compared with the fair proposal with a high stake size, we speculate that perhaps the contextual information (i.e., both the low and high stake sizes in this study) induced a negative emotional response to fair proposals with a low stake size and that this integrated information was reflected in the activation of the left dorsal-anterior insula. However, this explanation needs to be considered with caution because the interaction effect in the left dorsal-anterior insula disappeared when only the human condition rounds were analyzed. Therefore, the present study indicated that the dorsal-anterior insula responds to fair and unfair proposals differently depending on the size of the monetary stake, suggesting that the left dorsal-anterior insula may play a role in detecting social norm violations by integrating emotion and cognition in connection with specific contextual information.

Unlike the anterior insula, the modulation effects in the bilateral mid-posterior insula and the adjacent ventral insula were connected with decreased activity in response to the fair proposal with a high stake size. In the low stake size condition, the activation of the posterior insula was stronger for the fair proposals than for the unfair proposals. This finding is consistent with a previous study (Wright et al., 2011). A low stake size was used in that study in which Wright and his colleague found a negative correlation with inequality in the posterior insula (i.e., greater activation for a more equal allocation). These researchers used prediction error to explain their observation. That is, when the subjects formed predictions about inequality, an equal proposal led to a prediction error, which induced stronger activity in the posterior insula (Wright et al., 2011). We speculated that this seems true in the low stake size. However, in our study, in the high stake size condition, we found no differences between the fair and unfair proposals in the posterior insula, suggesting a modulation role of stake size on the activation of the posterior insula. In brief, the posterior insula may respond differently to fair and unfair proposals depending on the size of the monetary stake. This pattern is different from what we observed for the dorsal-anterior insula.

### Neural correlates of inter-individual differences in behavioral modulation effects

Money talks, but maybe not for everyone. Neural correlates of inter-individual differences in the deviation from fairness-related normative choices were also investigated in this study. Our data indicated that this behavioral inter-individual difference was associated with inter-individual differences in the modulation effects in the activation of the left IFG. When we differentiated the participants who showed a behavioral modulation effect from the whole group, the left IFG also showed a significant modulation effect. The neural correlates reflected that a participant who was more likely to make a decision that deviated from the fairness norm showed increased activation in the left IFG when facing an unfair proposal with a high stake size. A possible explanation for this neural correlate is that a large monetary temptation may induce a strong conflict between fairness and self-interest and thus require greater cognitive control. In the context of the current experimental design, the psychological process of cognitive control is likely to control emotion-driven prepotent responses to perceived unfairness, which could have led to a reduced rejection rate and a high level of activation for an unfair proposal with a high stake size. A core component of cognitive control—the ability to regulate thoughts and actions in accordance with internally represented behavioral goals—may be its intrinsic variability, which may be reflected in differences in the activations of the prefrontal cortices (Braver, 2012). Because the left IFG is also involved in cognitive control (Aron et al., 2004), during the UG individual differences in cognitive control may appear there. Additionally, a trend toward significance in the correlation between the modulation effects in the rejection rate and the modulation effects in the activation of the bilateral ACC (Table S1) supports our hypothesis that regions related to conflict and cognitive control participate in dealing with the conflict between fairness and self-interest resulting from a large monetary temptation.

Another possible explanation is that this neural correlate reflects inter-individual differences in emotional regulation. Previous studies have linked the IFG with emotion reappraisal (for a review, see Ochsner and Gross, 2005). A recent study further found that, when facing unfair proposals, the activation of the left IFG induced by emotional reappraisal was positively correlated with the ability to regulate emotion, as measured by an emotion regulation questionnaire (Grecucci et al., 2013). We speculate that some stable traits, such as reward sensitivity and personality, could account for our observed individual differences in norm enforcement, in line with the opinion that cognitive and personality factors may influence cognitive control (Braver, 2012). Previous studies have suggested that inter-individual differences in personalities (straightforwardness and trust) and central serotonin transmission account for individual differences in people's reaction to unfairness (Takahashi et al., 2012). Future studies need to further explore the psychological and neurobiological mechanisms underlying inter-individual differences in norm enforcement. An important reason why we should consider these inter-individual differences and their neural correlates is that aberrant social behavior has often been observed in psychiatric disorders (Kishida et al., 2010; Hasler, 2012), so investigating the neural correlates of individual differences in healthy participants may be helpful for understanding the neural basis of aberrant social behavior in psychiatric patients.

Taking into consideration all of the above results, we have provided neural evidence for the modulation of fairness by the size of a monetary incentive. By manipulating monetary incentives to alter the fairness-related normative decision, we have provided deeper insight into the neural substrates of the normal normative decision.

## Author contributions

All authors were involved in the design and implementation of the study and the writing of the manuscript. Authors Yuan Zhou and Shu Li devised the concept and supervised the study. Author Yun Wang collected the imaging data. Authors Yuan Zhou, Yun Wang, Li-Lin Rao, and Liu-Qing Yang carried out the analysis. Authors Yuan Zhou, Yun Wang, Li-Lin Rao, and Shu Li joined in the interpretation of data.

### Conflict of interest statement

The authors declare that the research was conducted in the absence of any commercial or financial relationships that could be construed as a potential conflict of interest.
