# Supplementary material for: Money talks: neural substrate of modulation of fairness by monetary incentives
Source: Front Behav Neurosci. 2014 May 5;8:150. doi: 10.3389/fnbeh.2014.00150 (PMC4017157; doi:10.3389/fnbeh.2014.00150)
Supplement: Supplementary file 1 [file Presentation1.PDF]

Supplementary materials

Supplementary text

Brain activations influenced by the interaction of fairness and stake size in the striatal subregions

Because the clusters also included the striatum, we divided the clusters into subregions by intersecting them with the striatal three subregions template (FSL template for striatum, see Figure S2A) (http://fsl.fmrib.ox.ac.uk/fsl/fslwiki/Atlases/striatumconn) in order to examine the simple effect of each striatal subregion.

We found that the modulation effects in the striatal subregions were similar to each other. In general, the activations in the striatal subregions were stronger for unfair proposals than for fair proposals when the stake size was high, and the activation in the striatal subregions was weaker for unfair proposals than for fair proposals when the stake size was low. Specifically, the activation level in the bilateral sensorimotor, left executive and left limbic subregions was significantly stronger for unfair proposals than for fair proposals when the stake size was high ($ps < .05$), and these effects in the right executive and right limbic subregions were marginally significant ($ps < .1$). The activation in the right limbic subregion was significantly stronger for fair proposals than for unfair proposals when the stake size was low ($p = .028$), and these effects in the right sensorimotor and right executive subregions were marginally significant ($ps < .1$), but these effects in the left subregions were not significant (Figure S2B).

In addition, similar modulation effects were found in the human condition according to the striatal subregions template. Specifically, the activations in the right sensorimotor and left executive subregions were significantly stronger for unfair proposals than for fair proposals when the stake size was high ($ps < .05$), but these effects in the right executive and bilateral limbic subregions were not significant. The activations in the right executive and bilateral limbic subregions were significantly stronger for fair proposals than for unfair proposals when the stake size was low ($ps < .05$), but these effects in the right sensorimotor and left executive subregions were not significant (Figure S2C).

Neural correlates of inter-individual differences in the behavioral modulation effect

When we combined the trials from the human proposals with those from computer proposals, similar but weaker negative correlations were found in the bilateral IFG (left IFG: MNI coordinate = [-45 33 12]; right IFG: MNI coordinate = [42 12 24]; $p < .005$, voxel size > 10 voxels) (Figure S3). To consider the inter-individual differences, we analyzed those participants who showed a behavioral modulation effect (n=16). When we extracted the value of the mean of the interaction effect, both clusters showed a significant interaction effect (left IFG: $t = 2.79$, $p = .014$; right IFG: $t = 3.35$, $p = .004$).

Supplementary discussion

Fairness-related neural networks

As in prior studies (Corradi-Dell'Acqua et al., 2013; Dulebohn et al., 2009; Güroğlu et al., 2010; Güroğlu et al., 2011; Guo et al., 2013; Sanfey et al., 2003), unfair proposals activated the DLPFC, insula, ACC and striatum more than fair proposals, suggesting that these regions are related to the perception of unfairness and suggesting that these regions constituted a neural network engaged in the emotional, cognitive and motivational processes associated with (un)fairness.

Furthermore, we found that fair proposals activated the MPFC in contrast to unfair proposals. The MPFC is also known to be involved in fairness processing (Tabibnia et al., 2008) and is often involved in inferring another's intention (Van Overwalle, 2009). The activation of the MPFC when facing fair proposals suggests a mental process in which participants are inferring the proposer's intention about why they would make an equal split when maximizing the proposer's financial interest would be rational.

Supplementary tables and figures

Table S1 Regions whose BOLD signal change, as detected from the [high$_{(unfair-fair)}$-low$_{(unfair-fair)}$] contrast, varied with the modulation effect on the rejection rate in the human condition (*p* < .005, 10 voxels) *cluster-level correction *p* < .05

Cluster size	Hemisphere	Brain region	BA	MNI coordinates	Peak T value
53	right	inferior frontal gyrus	47	51 18 -9	4.61
89	bilateral	anterior cingulate	24	3 30 18	4.65
30	right	middle frontal gyrus	8	27 21 45	3.62
177	left	inferior frontal gyrus*	44	-51 15 15	4.24
20	left	middle frontal gyrus	10	-39 48 18	3.39
10	left	cingulate gyrus	32	-3 15 42	3.38
21	right	inferior frontal gyrus	45	42 9 24	3.35
17	right	superior frontal gyrus	8	6 33 51	3.18
15	right	middle frontal gyrus	9	42 21 24	3.16

Table S2 Interaction effect of fairness with stake size in the participants who showed a behavioral modulation effect in the human condition (n=17) (p < .005, 10 voxels)

Cluster size	Hemisphere	Brain region	BA	MNI coordinates	Peak T value
51	right	insula	13	42 -12 12	3.96
26	left	insula	13	-42 -12 3	3.91
25	right	inferior frontal gyrus	45	63 24 15	3.9
23	left	inferior frontal gyrus	46	-57 27 12	3.74
24	right	inferior frontal gyrus	44	39 6 27	3.79
16	left	inferior frontal gyrus	44	-57 15 21	4.04
12	right	superior frontal gyrus	8	15 39 48	3.59

Table S3 Insular coordinates reported in previous studies using the ultimatum game

Number	Author and year	Contrast	MNI coordinate		
			x	y	z
1	Sanfey et al., 2003	unfair>fair(human condition)	35	15	4
			-33	14	0
2	Tabibnia et al., 2008	accepted < rejected (idiographically defined unfair offers)	-34	20	-6
			32	22	-10
		accepted< rejected (unfair offers)	-28	8	-6
		rejected(ideographically defined unfair offers)>baseline	36	18	-8
3	Chang et al., 2009	positive expectation deviation(offered more than expected)	-42	12	-4
			42	16	-8
		negative expectation deviation(offered less than expected)	-38	-12	-2
			40	-12	-10
4	Halko et al., 2009	small offers > large offers (non-competition trials)	42	21	-4
		small offers > large offers (competition trials)	35	25	-7
			-32	21	4
		non-competition > competition (rejected offers)	49	-4	4
5	Güroğlu et al., 2010	response × context	45	24	-12
			-33	21	6
			39	-12	15
6	Güroğlu et al., 2011	response × intentionality	42	24	-6
			-36	15	-9
7	Wright et al., 2011	Inequality: negative main effect across all trial types	32	-21	21
			42	-24	24
		Inequality: negative main effect in matched trials (M-in-H and M-in-L)	-45	-18	0
			-36	-12	3
			-54	-12	6
		Inequality: interaction with social context (M-in-Land M-in-H)	48	-3	9
			36	6	9
			-48	-9	12
8	Kirk et al., 2011	controls unfair > fair	32	20	-8
			-44	24	8
		meditators unfair > fair	-36	4	8
			32	-24	16
			-32	-24	16

		meditators unfair> controls unfair	44	-4	8
			-44	-4	8
			36	-28	20
			-44	-24	20
9	Gospic et al., 2011	unfair > fair (placebo condition)	-30	24	3
		unfair > fair (oxazepam condition)	36	21	12
10	Kim et al., 2012	significant correlation with lateral OFC biasing decisions	-33	15	-3
		significant functional connectivity between lateral OFC and bilateral insula	-42	12	-9
			48	9	-15
11	Harle et al., 2012	mood condition (sad/neutral) × offer amount	39	30	-8
			-33	12	-16
12	Harle et al., 2012	effect of offer fairness(unfair > fair) × age group	-35	6	-9
			42	15	-5
13	Grecucci et al., 2013	unfair offer rejected Up>Look	-45	5	10
			36	17	4
		unfair rejected offers for Down<Look<Up	-45	-22	25
			27	20	7
			-36	11	4
14	Corradi-Dell’Acqua et al., 2013	ultimatum game>free win	30	22	2
			-34	16	0
		rejected>accepted ultimatum game offer	-36	16	-4
15	Guo et al., 2013	unfair > fair	-30	24	6
			32	28	4
		rejected > accepted unfair offers	-36	16	4
			32	16	12
		rejected > accepted unfair losses	-34	18	14
			34	12	12

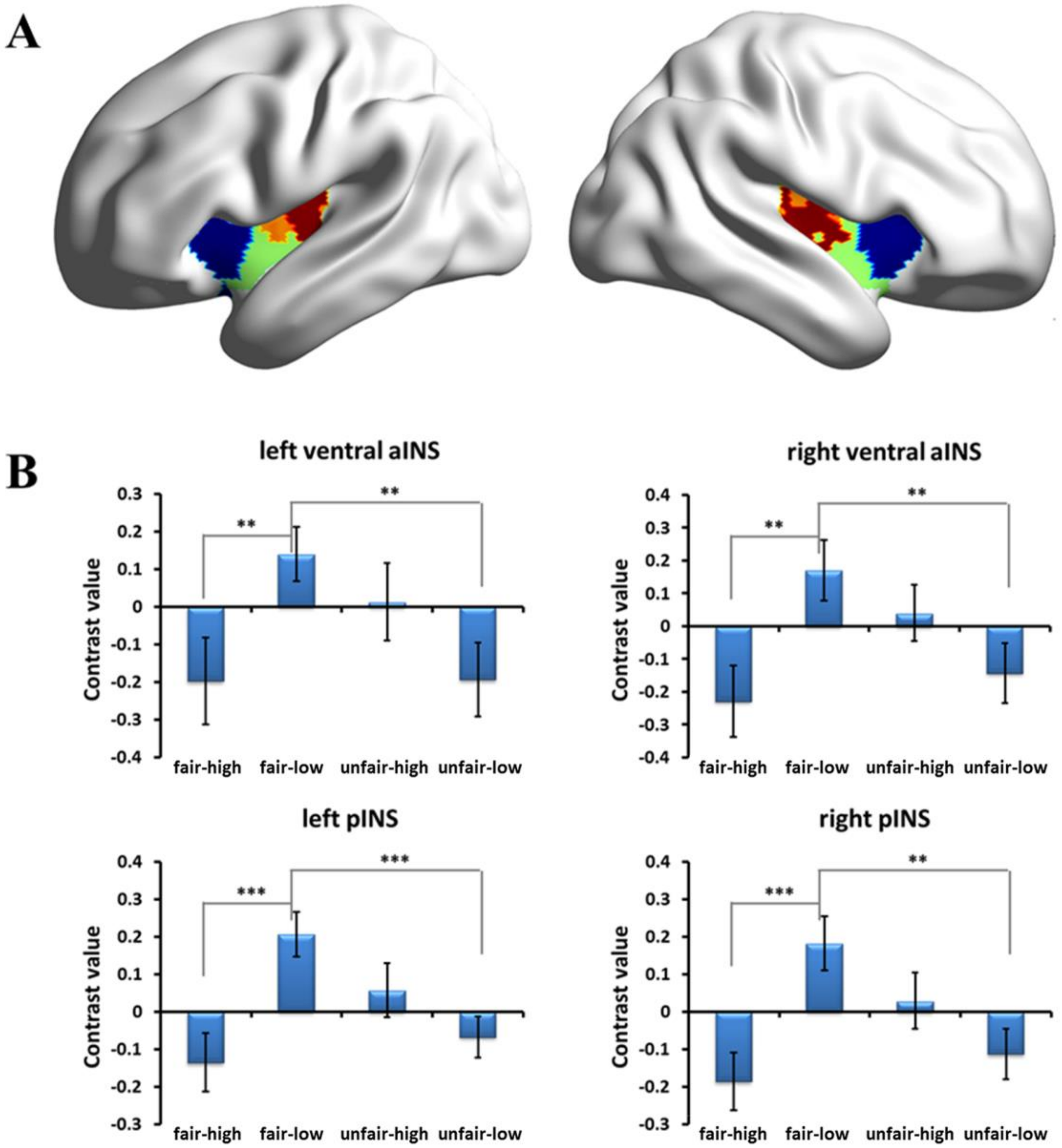

Figure S1 Insular subregions influenced by the interaction between fairness and stake size in the human proposer condition. (A) Insular subregions intersected by Kelly's template. (B) Simple effects of the modulation of fairness by stake size in insular subregions. **p<0.01,***p<0.001.

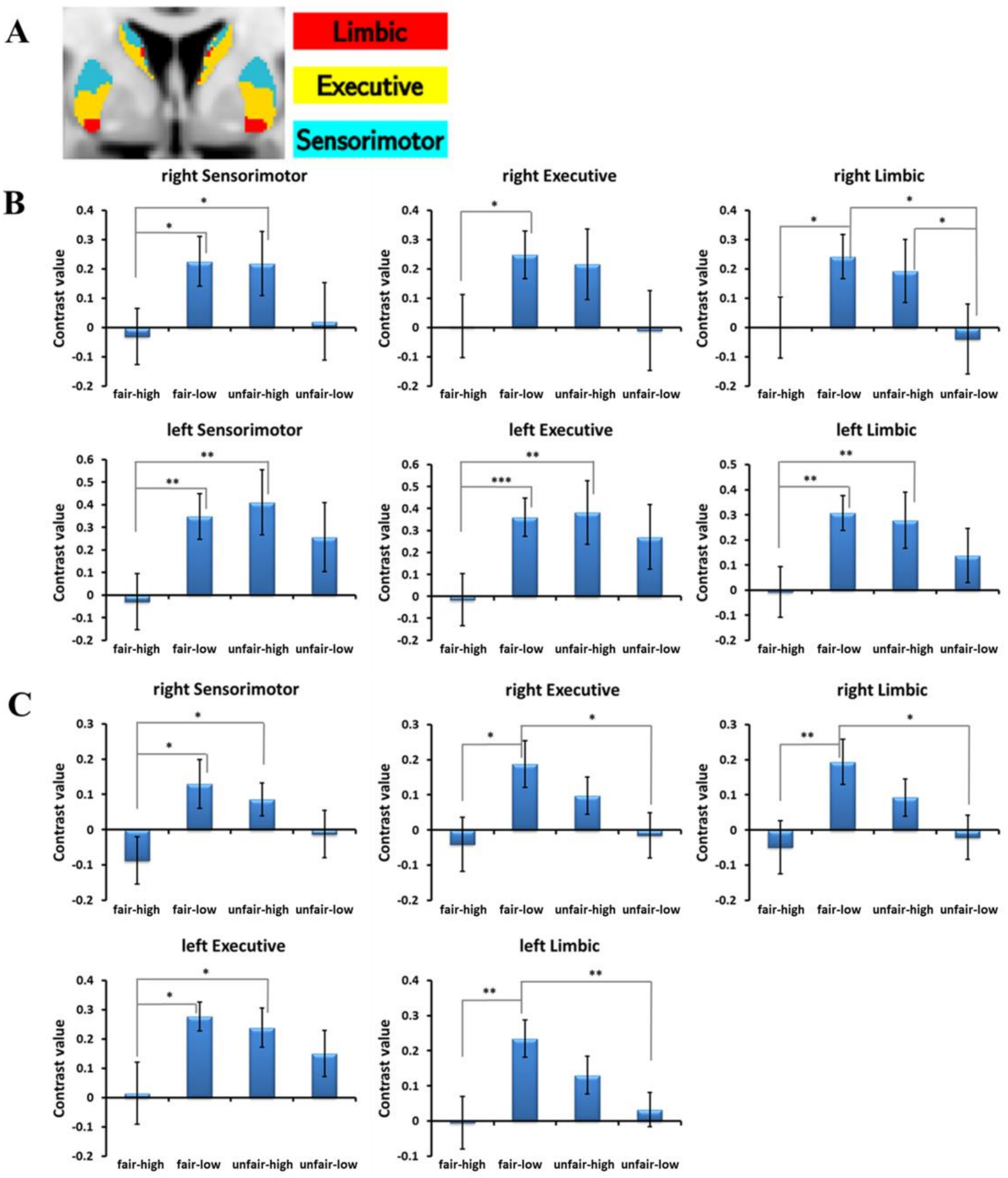

Figure S2 Striatal subregions influenced by the interaction between fairness and stake size. (A) Striatal subregion atlas (http://fsl.fmrib.ox.ac.uk/fsl/fslwiki/Atlases/striatumconn). (B) Simple effects of the modulation of fairness by stake size in the striatal subregions for all the proposals. (C) Simple effects of the modulation of fairness by stake size in the striatal subregions for the human proposals. *p<0.05, **p<0.01,***p<0.001.

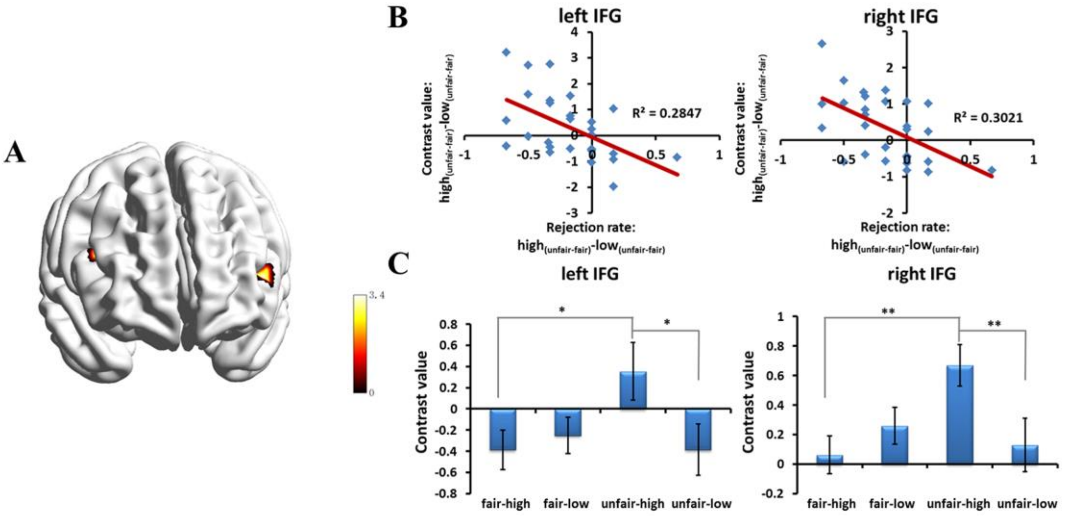

Figure S3 Neural correlates of inter-individual differences in the behavior modulation effect of the rejection rate. (A) Regions showing the neural correlates of inter-individual differences in the behavior modulation effect of the rejection rate for all the proposals. (B) Significant negative correlations were found in the bilateral IFG between the bold signal of the [high$_{(unfair-fair)}$-low$_{(unfair-fair)}$] contrast and the rejection rate of the [high$_{(unfair-fair)}$-low$_{(unfair-fair)}$] proposals. (C) Significant interaction effect of fairness*stake size was found in the bilateral IFG ($p < .005$, 10 voxels). *p<0.05, **p<0.01.
